# Concentration Dependence of Optical Properties of Double-Doped LiTaO_3_:Cr^3+^:Nd^3+^ Crystals

**DOI:** 10.3390/ma18143218

**Published:** 2025-07-08

**Authors:** Nikolay V. Sidorov, Lyubov A. Bobreva, Alexander Yu. Pyatyshev, Mikhail N. Palatnikov, Olga V. Palatnikova, Alexander V. Skrabatun, Andrei A. Teslenko, Mikhail K. Tarabrin

**Affiliations:** 1 Tananaev Institute of Chemistry—Subdivision of the Federal Research Centre, Kola Science Centre of the Russian Academy of Sciences (ICT RAS), 184209 Apatity, Murmansk Region, Russia; n.sidorov@ksc.ru (N.V.S.); l.bobreva@ksc.ru (L.A.B.); m.palatnikov@ksc.ru (M.N.P.); o.palatnikova@ksc.ru (O.V.P.); 2 P. N. Lebedev Physical Institute of the Russian Academy of Sciences, 119991 Moscow, Russia; skrabatunav@lebedev.ru; 3 Physics Department, Bauman Moscow State Technical University, 105005 Moscow, Russia; 4 Infrared Laser Systems Laboratory, Bauman Moscow State Technical University, 105005 Moscow, Russia; aateslenko@gmail.com (A.A.T.); tarabrinmike@yandex.ru (M.K.T.)

**Keywords:** lithium tantalate, double doping with Cr and Nd, X-ray analysis, conoscopic pattern, optical absorption, Raman scattering, hydrogen bond, stretching vibrations of OH^−^ groups, point structural defects, complex defects

## Abstract

LiTaO_3_ crystals doped with Cr^3+^ and Nd^3+^ ions are promising for developing active nonlinear laser media. In this work, the defect structure of LiTaO_3_ crystals, including those doped with Cr^3+^ and Nd^3+^, is examined. X-ray patterns of all six investigated LiTaO_3_:Cr:Nd crystals are identical and correspond to a highly perfect structure. Using optical microscopy, the presence of defects of various shapes, microinhomogeneities, and lacunae was revealed. The optical absorption and Raman scattering spectra of a series of nonlinear, optical, double-doped LiTaO_3_:Cr^3+^:Nd^3+^ (0.06 ≤ [Cr^3+^] ≤ 0.2; 0.2 ≤ [Nd^3+^] ≤ 0.45 wt%) crystals showed that at concentrations of doping Cr^3+^ ions less than 0.09 wt% and Nd^3+^ ions less than 0.25 wt%, the crystal structure is characterized by a low level of defects, and the optical transmission spectra characterized by narrow lines corresponding to electron transitions in Nd^3+^ ions. In this case, for the radiative transition in the cation sublattice, the existence of three nonequivalent neodymium centers is observed, and for the radiative transition, two nonequivalent centers are observed. IR absorption spectroscopy in the OH^−^-stretching vibration range revealed two main spectral regions: 3463–3465 cm^−1^, associated with stoichiometry changes, and 3486–3490 cm^−1^, linked to complex defects such as (V^-^_Li_)-OH and (Ta^4+^_Li_)-OH. A distinct low-intensity line at ~3504 cm^−1^ was observed only in doped crystals, attributed to (Nd^2+^_Li_)-OH defects that significantly distort the oxygen-octahedral clusters due to the larger ionic radius of Nd^3+^ compared to Ta^5+^. In contrast, Cr-related defects cause only minor distortions. The Klauer method indicated that the highest concentration of OH^−^-groups occurs in the LiTaO_3_:Cr^3+^ (0.09 wt%):Nd^3+^ (0.25 wt%) crystal, where multiple complex defects are present.

## 1. Introduction

The features of the defect structure of lithium tantalate crystal (LiTaO_3_), as a non-stoichiometric oxygen-octahedral phase of variable compositions with a wide homogeneity region in the phase diagram (46.0–50.4 mol% Li_2_O), make it possible to effectively regulate the physical characteristics of functional materials based on it by doping or changing the stoichiometry (ratio R = [Li]/[Ta]) [1,2,3,4]. LiTaO_3_:Cr^3+^, LiTaO_3_:Nd^3+^, and especially double-doped LiTaO_3_:Cr^3+^:Nd^3+^ crystals are promising for the development of functional materials for active nonlinear laser media when laser generation of radiation at a certain frequency and nonlinear optical conversion at this frequency simultaneously occur in a single crystal, as well as for the development of compact functional materials generating coherent radiation for laser printing, for information storage and recording devices, and for optical communications [5,6,7,8,9,10,11,12]. Double doping of LiTaO_3_ and isomorphic LiNbO_3_ crystals enables more precise adjustment of the cation sublattice’s structural ordering compared to single doping. This process also alters the polarizability and dipole moment of the MeO_6_ clusters (which include Li^+^, Ta^5+^, Nb^5+^, the alloying element, and vacant octahedra), thereby influencing the materials’ ferroelectric and nonlinear optical properties [13,14,15,16,17]. At the same time, the combined use of transition (Cr^3+^) and rare earth (Nd^3+^) metals for doping allows for an increase in the quantum yield of luminescence and the efficiency of energy conversion into laser radiation due to the synergistic effect [8,18,19,20,21,22,23,24,25,26,27,28,29,30,31,32,33,34], including for organic compounds [35]. For the first time, laser generation using the Nd^3+^-Cr^3+^ pair was realized in crystals of yttrium aluminum garnet YAG:Cr:Nd [36], chromium–neodymium–aluminoborate (Nd(Al,Cr)_3_(BO_3_)_4_ [37], and gadolinium–scandium–gallium garnet doped with chromium and neodymium [38]. However, the Nd^3+^-Cr^3+^ pair requires careful selection of the optimal concentration of doping elements in the crystal, which is a technologically difficult task when growing a single crystal of a strictly specified composition and requires research into the influence of the doping elements Nd^3+^ and Cr^3+^ on the concentration dependences of the optical characteristics of laser materials.

In [39], the optical absorption spectra and luminescence spectra of two LiTaO_3_:Cr^3+^:Nd^3+^ single crystals with different neodymium ion contents (0.2 and 0.4 wt%) but with the same chromium ion content (0.1 wt%) were investigated for the first time. Two spin-allowed bands of chromium transitions, ^4^A_2_-^4^T_1_ and ^4^A_2_-^4^T_2_, were found in the absorption spectrum, as well as a band corresponding to the forbidden transition ^4^A_2_-^2^E and a number of lines corresponding to transitions in neodymium ions. A number of lines corresponding to transitions in Cr^3+^ and Nd^3+^ ions were also observed in the luminescence spectra. The excited-state lifetime of the Cr^3+^ ion was also determined, establishing the occurrence of nonradiative energy transfer to the Nd^3+^ ion. Thus, in [38], it was shown that the LiTaO_3_:Cr^3+^:Nd^3+^ crystal is a promising active nonlinear laser medium. However, such a high concentration of Cr^3+^ ions in the crystal (0.1 wt%) leads to very strong absorption of laser radiation by the crystal. Also, for a single crystal of only one composition LiTaO_3_:Cr^3+^(0.2):Nd^3+^(0.45 wt%), upon excitation by a laser line of the near IR range of 785 nm [40], the Raman spectra in polarized radiation were studied.

A significant limiting factor influencing the synergetic effect is the set of point defects of the crystal in the form of doping ions, main ions (Li^+^ and Ta^5+^) located in positions other than their own, as well as complex defects (V_Li_)-OH, (Ta_Li_)-OH, etc., caused by the presence of hydrogen bonds in the structure of the LiTaO_3_ crystal. The effect of complex defects caused by the presence of hydrogen bonds on the physical characteristics of LiNbO_3_ crystals of different compositions has been studied in detail in dozens of works (see reviews [41,42,43,44]). At the same time, for LiTaO_3_ crystals of different compositions, there are fewer studies in the literature devoted to complex defects caused by the presence of OH^−^ groups and their effect on physical properties [13,45,46,47,48,49,50,51,52]. The presence of hydrogen in the structure of the LiTaO_3_ crystal can influence not only the quantum yield of luminescence and the efficiency of energy conversion into laser radiation, but also practically significant properties such as photoluminescence, photoinduced change in the refractive index, hologram stability, dark conductivity, etc.

The quasi-elastic constant of an O–H bond, which determines its OH^−^ stretching frequency, varies depending on the specific MeO_6_ octahedral cluster in LiTaO_3_ and LiNbO_3_ crystals, as well as on the OH^−^ group’s position within that cluster. The frequency of the line corresponding to the stretching vibrations of the OH^−^ group is determined by the quasi-elastic constant of the hydrogen bond. This constant, in turn, depends on the configuration of the MeO_6_ cluster, which is influenced by the crystal’s stoichiometry as well as the type and concentration of the alloying elements [41,42,43,44]. The stretching vibrations of the hydrogen atoms of the OH^−^ groups are manifested both in the Raman spectra and in the IR absorption spectra. However, the IR absorption spectra are much more sensitive to the presence of OH^−^ groups than the Raman spectra, due to the very strong absorption of infrared radiation.

For the LiTaO_3_:Nd^3+^(0.1 wt%) crystal, it was found [53] that annealing the crystal at a temperature of 980 K leads to a shift in hydrogen ions relative to the TaO_6_ groups and a decrease in absorption. In this case, changes occur in the IR absorption spectrum in the region of stretching vibrations of the OH^−^ groups, while the absorption maximum shifts by 8 cm^−1^ toward lower frequencies. In the IR absorption spectrum of LiNbO_3_:Nd^3+^(0.2 wt%), LiTaO_3_:Nd^3+^(0.5 at%), and LiTaO_3_:Cr crystals, an absorption line with a frequency of 3486 cm^x^, consisting of one component, was recorded [54,55]. The authors of [56] discovered an asymmetric absorption line with a frequency of 3484 cm^−1^ in the IR absorption spectrum of the LiTaO_3_:Nd^3+^ crystal, corresponding to the stretching vibrations of the OH^−^ groups, as well as a number of narrow lines corresponding to transitions in the neodymium ion. Earlier in [57], the IR absorption spectra of two crystals, LiNbO_3_:Cr^3+^(0.075):Nd^3+^(0.051 at%):Mg and LiNbO_3_:Cr^3+^(0.25):Nd^3+^(0.074 at%):Mg, were analyzed. For these crystals, two absorption lines were recorded with frequencies of 3506 (which corresponds to the Cr^3+^(Nb^5+^)-OH^−^-Mg^2+^(Li^+^) complex) and 3522 cm^−1^ (which corresponds to the Nd^3+^(Li^+^)-OH^−^-Mg^2+^(Nb^5+^) complex). The addition of neodymium ions to the LiNbO_3_:Yb^3+^ crystal resulted in a slight decrease in the absorption coefficient in the region of stretching vibrations of the OH^−^ groups [58]. With an increase in the Nd^3+^ content from 0.44 mol% to 0.85 mol%, the OH^−^ group stretching vibration band shifts by 1 cm^−1^, and two absorption maxima merge into one in the LiNbO_3_:Nd^3+^ crystal [59]. For LiNbO_3_:Cr^3+^(0.1, 0.25 mol%) crystals, the absorption line in the region of OH^−^ group stretching vibrations is located near 3484 cm^−1^ [60]. Increasing the chromium concentration to 0.5 mol% shifts the absorption line to 3490 cm^−1^. Therefore, the data from the literature on the absorption and vibrational spectra in LiTaO_3_:Cr^3+^:Nd^3+^ crystals are very limited.

In this work, we analyze data from X-ray diffraction, optical microscopy, and conoscopic patterns, and investigate the concentration dependence of the optical transmission, Raman scattering, and IR absorption spectra in the region of OH^−^ group stretching vibrations of a series of several LiTaO_3_ crystals doped with Cr^3+^ and Nd^3+^ in the following concentrations—LiTaO_3_:Cr^3+^(0.06):Nd^3+^(0.20 wt%), LiTaO_3_:Cr^3+^(0.07):Nd^3+^(0.28 wt%), LiTaO_3_:Cr^3+^(0.09):Nd^3+^(0.25 wt%), LiTaO_3_:Cr^3+^(0.1):Nd^3+^(0.25 wt%), LiTaO_3_:Cr^3+^(0.11):Nd^3+^(0.41 wt%), and LiTaO_3_:Cr^3+^(0.2):Nd^3+^(0.45 wt%)—were investigated. These crystals show promise for the development of active nonlinear laser media. In the studied samples, chromium and neodymium ions are present in the trivalent state. Nominally pure LiTaO_3_ crystals of congruent composition and LiTaO_3_:Cr^3+^(0.005 wt%) crystals were used as comparison samples in the studies.

## 2. Materials and Methods

Growing optically and compositionally homogeneous LiTaO_3_ crystals with double doping is a non-trivial technological task. Doping additives (Cr and Nd) have different impurity distribution coefficients K_D_. Consequently, the melt composition near the crystallization front and the doped crystal composition from the cone to the end can change. This leads to a decrease in its compositional and optical homogeneity, and the crystal characteristics can differ throughout its volume. To minimize such effects, natural changes in the growth parameters for the Czochralski crystal growth process were used: the speed of rotation and movement of the crystal, the temperature gradient in the melt and growth zone, and a combination of these parameters. This work employed a specially designed heating unit and controlled crystallization rates, along with careful melt preparation (including overheating) prior to crystal growth. In addition, long-term, post-growth annealing and appropriate electrothermal treatment conditions were applied to achieve single-domain formation in the crystals. In addition, when growing LiTaO_3_:Cr:Nd crystals, no more than 20% of the melt volume crystallized.

**Figure 1:**
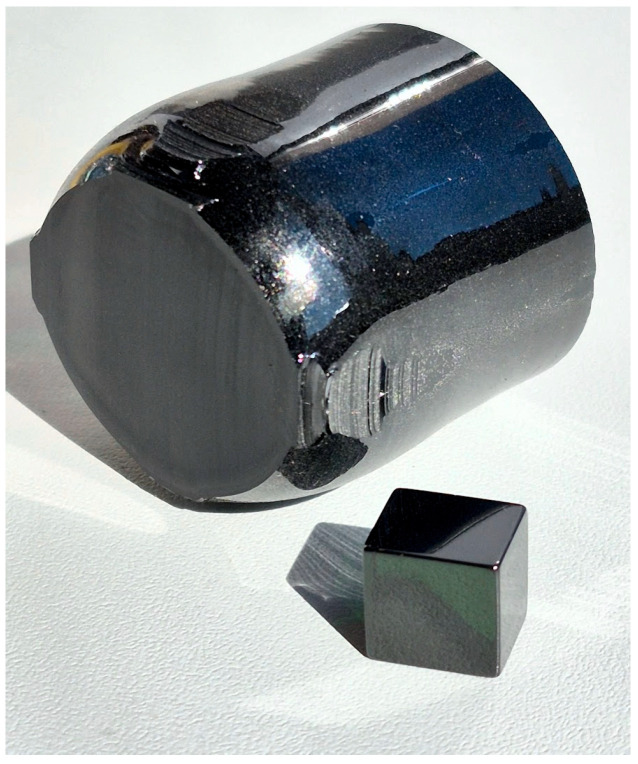
Grown LiTaO_3_:Cr:Nd single crystal and a sample for research in the form of a cube with an edge length of 5 mm.

The studied LiTaO_3_, LiTaO_3_:Cr, and LiTaO_3_:Cr:Nd crystals were grown in an argon atmosphere by the Czochralski method. The crystals were grown from a Pt/Rh10 ∅ 80 mm crucible under conditions of an average axial gradient of ~12 °C/cm in the X-axis direction (X-cut) at rotation (~14 rpm) and translation (~2 mm/h) speeds (see Figure 1). In this case, the crystal increment rate was ~2.6–2.7 mm/h. The crystals were grown on a growth setup Kristall-2 (Voroshilovgradsky zavod electronnogo mashinostroeniya, Voroshilovgrad, USSR) equipped with an automatic crystal diameter control system. A total of eight crystals were grown: one LiTaO_3_ crystal, one LiTaO_3_:Cr crystal, and six LiTaO_3_:Cr:Nd crystals (see Table 1).

For the synthesis of the LiTaO_3_ charge of congruent composition, ultra-pure tantalum pentoxide Ta_2_O_5_ and high-purity lithium carbonate Li_2_CO_3_ with a concentration of foreign impurities at the level of <3·10^−4^ wt% (JSC Solikamsk Magnesium Plant, Solikamsk, Russia) were used. Cr_2_O_3_ and Nd_2_O_3_ oxides with an impurity concentration of <5·10^−4^ wt% (LLC NevaReaktiv, St. Petersburg, Russia) were introduced into the mixture as alloying additives.

The concentration of Cr and Nd in LiTaO_3_:Cr:Nd crystals was determined by atomic emission spectrometry on an ICPE 9000 spectrometer (Shimadzu, Kyoto, Japan) with an accuracy of 4·10^−3^%.

A batch of congruent composition (R ≈ 0.92) was synthesized from these initial components. The impurity composition of the LiTaO_3_ batch and one of the grown LiTaO_3_:Cr:Nd crystals (LT-5 crystal), which was determined using the spectral analysis method, is given in Table 2. The impurity composition values of the remaining LiTaO_3_:Cr:Nd crystals are similar and therefore are not given in Table 2. This table also provides the Curie temperature (T_C_) value.

The growth process was completed when the weight of LiTaO_3_:Cr:Nd crystals reached ~450–470 g. The growth process parameters were selected based on the need to obtain a flat crystallization front, which should ensure a sufficiently high structural perfection of the crystal and was achieved by experimentally selecting the pulling speed, the rod rotation speed, and the temperature gradient at the crystallization front. The grown LiTaO_3_:Cr:Nd crystals had a flat or slightly convex crystallization front, diameter of ~34–38 mm, and a cylindrical part length of ≈38–40 mm. Doping impurities were introduced into the batch in the form of oxides with subsequent thorough mixing. Before the start of crystal growth, the melt was held for 8 h under conditions of overheating by ~70 °C relative to the melting point (T_m_ ≈ 1650 °C) of the LiTaO_3_ crystal for homogenization of the impurity in the melt. After growth, LiTaO_3_:Cr:Nd crystals were annealed at 1400 °C in a growth setup for 10 h and then cooled at a rate of ~50 °C/h. Long post-growth annealing is required to homogenize the composition of the doped crystal and relieve thermal and mechanical stresses.

For spectral studies, samples in the form of 4 × 6 × 7 mm^3^ parallelepipeds were cut from a single-domain, single-crystal boule. The edges of the parallelepipeds coincided in direction with the direction of the main crystallographic axes X, Y, and Z. The faces of the parallelepipeds were carefully polished (the roughness was 0.026 μm).

X-ray phase analysis of LiTaO_3_:Cr:Nd single crystals was performed using an XRD-600 diffractometer from Shimadzu (Kyoto, Japan). To study the macro- and microstructure of LiTaO_3_:Cr:Nd crystals, a Thixomet image analysis system was used, including an Axio Observer. Dlm optical microscope from Karl Zeiss, coupled via a PixelLink PL-B774U digital video camera to a computer equipped with the ThixometPRO program. The samples for the studies were in the form of polished plates subjected to chemical etching at room temperature for 20 h in a mixture of mineral acids HF:HNO_3_ = 1:3. The setup for studying conoscopic images and the experimental technique are described in detail in [1]. Single-crystal plates with a thickness of 1 mm were used for the studies. The experiments were carried out using the second harmonic of the YAG:Nd^3+^ laser MLL-100 (Changchun New Industries Optoelectronics Tech. Co., Ltd., Changchun, China). The optical transmission spectra of single crystals were recorded using a UNICO 2800 UV/VIS spectrophotometer (United Products & Instruments, Dayton, NJ, USA) in the range of 190–1100 nm with a resolution of 0.2 nm. The measurements were performed at room temperature. The Raman spectra were recorded using a BWS465-532S i-Raman Plus spectrometer (B&W Tek, Plainsboro Township, NJ, USA) with a recording range of 65–4200 cm^−1^, equipped with a continuous laser with a wavelength of 532 nm. The laser radiation power during spectra recording was 30 mW, and the numerical aperture of the focusing system was ≈0.22. The laser spot size at the focus was 85 μm. All spectra were recorded at room temperature using backscattering geometry. In order to minimize the local influence of the exciting laser radiation, the optimal radiation focusing modes in the crystals under study and the useful signal accumulation time were selected in each experiment. The spectrometer resolution was 4.5 cm^−1^. The spectra were recorded using a Bruker VERTEX 70x FTIR spectrometer (Bruker, Karlsruhe, Germany) with a spectral resolution of 0.4 cm^−1^. Measurements were averaged over 50 scans for background and samples. The measurements were carried out taking into account the reflection of all elements and the absorption of the optical path. The photometric accuracy of the spectrometer used is better than 0.1%. The measurements were carried out in a vacuum at a pressure of 1.78 hPa and room temperature.

## 3. Results and Discussion

### 3.1. X-Ray Phase Analysis

**Figure 2:**
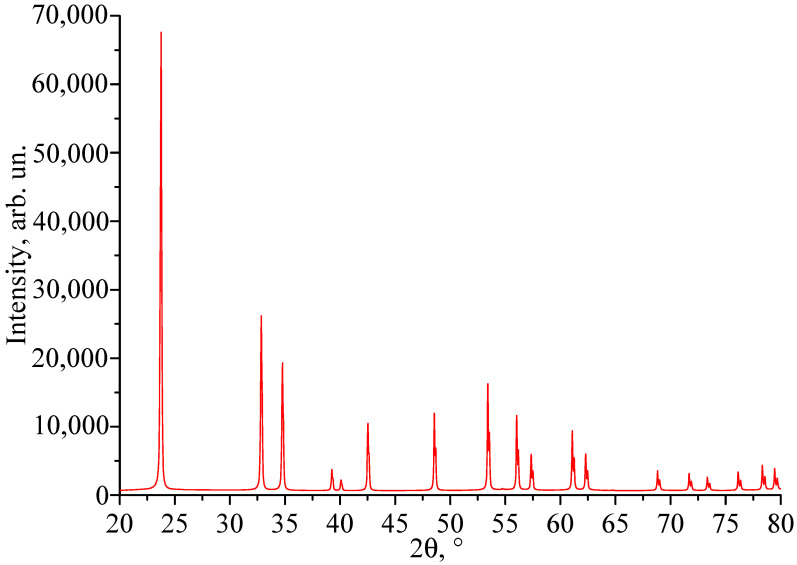
X-ray diffraction pattern of LT-8 crystal.

The performed X-ray phase analysis showed that the X-ray diffraction patterns of all six studied LiTaO_3_:Cr:Nd crystals are identical and correspond to a highly perfect structure of the LiTaO_3_ crystal, the unit cell of which is characterized by the space symmetry group $C_{3V}^{6}$ (R3c). Reflections corresponding to impurity phases were not detected. As an example, Figure 2 shows the X-ray diffraction pattern of the LiTaO_3_:Cr(0.2):Nd(0.45 wt%) crystal—LT-8—which is characterized by the highest concentration of chromium and neodymium and, therefore, has the most disordered structure. The unit cell parameters of the LT-8 crystal (Å): a = 5.16231; b = 5.16231; c = 13.77299; α = 90.000°; β = 90.000°; γ = 120.000°. The volume of the unit cell is 317.868 Å^3^.

### 3.2. Macro- and Microdefect Structures of LiTaO_3_:Cr^3+^:Nd^3+^ Crystals

**Figure 3:**
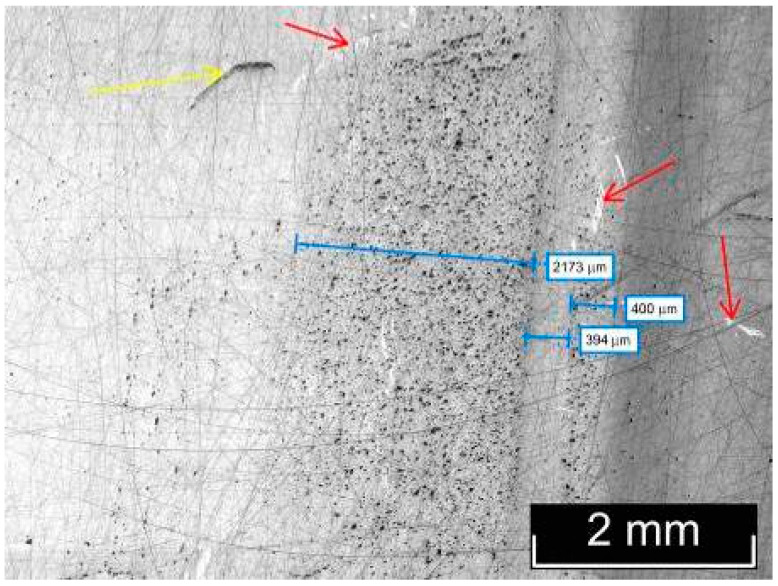
Macro- and microstructures of the Z-face of a LiTaO_3_:Cr:Nd crystal sample measuring 7 × 9 mm^2^, studied in the BF mode.

**Figure 4:**
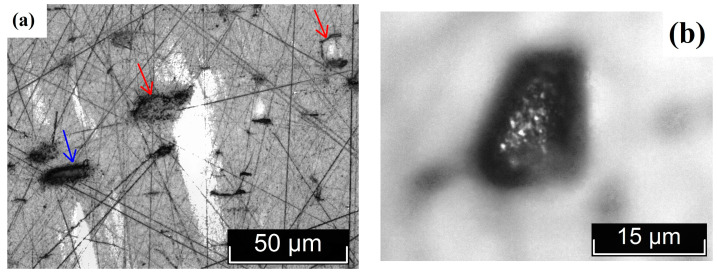
Microinhomogeneities located in the region of the stripes shown in Figure 3, against the background of internal hidden defects—(**a**); lacuna—(**b**).

**Figure 5:**
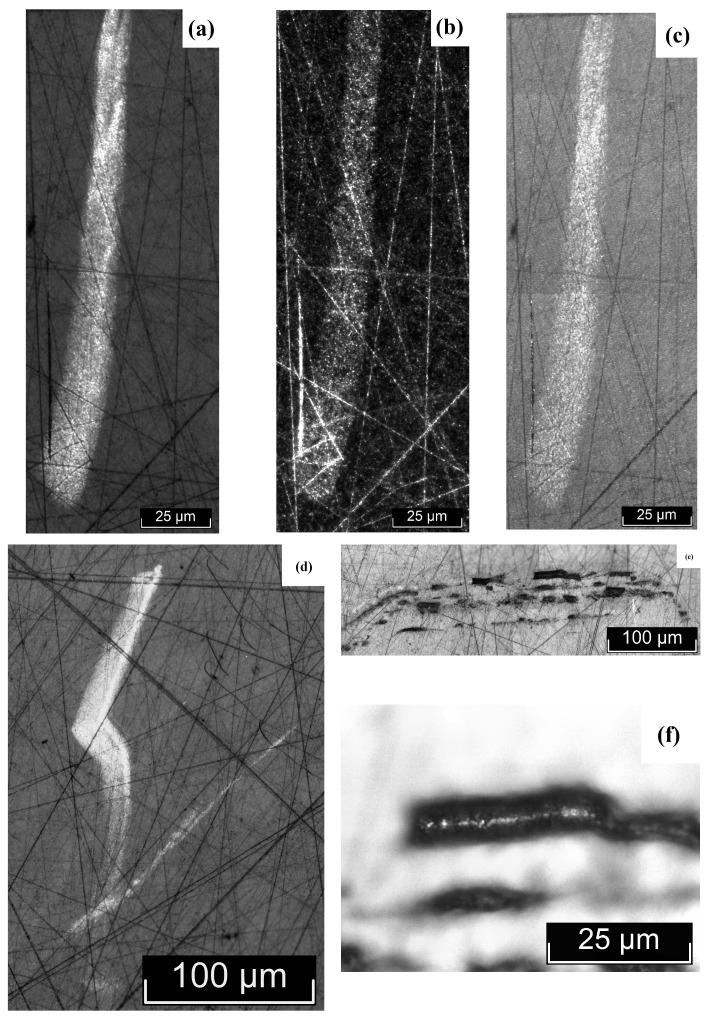
Internal defects of the Z-face of the LiTaO_3_:Cr:Nd crystal, recorded in the BF (**a**,**d**,**e**), DF (**b**), and DIC (**c**) modes. Hidden defects (**a**–**d**) and a defect emerging on the surface (**e**) and its structure (**f**).

**Figure 6:**
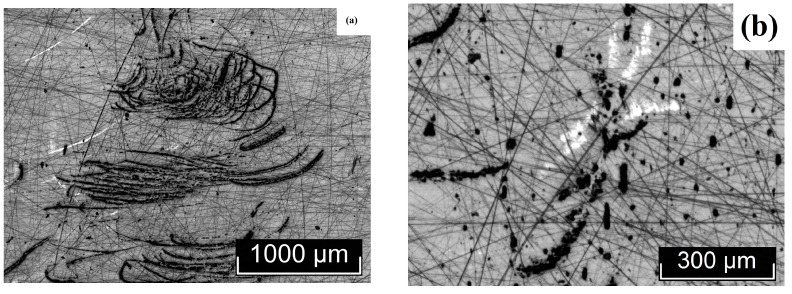
Macro- (**a**) and microstructures (**b**) of the X-face of a LiTaO_3_:Cr:Nd crystal sample measuring 9 × 8 mm^2^, studied in the BF mode.

The macro- and microstructures of LiTaO_3_:Cr:Nd crystals were investigated by us using optical microscopy methods, using the LT-5 crystal as an example. The most typical macro- and microstructural defects, inherent to varying degrees in all the studied LiTaO_3_:Cr:Nd crystals, are shown in Figure 3, Figure 4, Figure 5 and Figure 6. Image analysis showed that for all studied LiTaO_3_:Cr:Nd crystals, the defect structure is qualitatively and quantitatively similar. Thus, the macrostructure of the Z-face of a 7 × 9 mm^2^ sample investigated in the bright field (BF) mode is shown in Figure 3.

The image clearly shows two bands with a width of ~2.0 and 0.4 mm. These bands differ in hardness from the main part of the crystal, since under the same impact during sample preparation, the band areas slightly rise above the main surface (Figure 3). Apparently, these areas of the crystal have an increased content of alloying impurities and microinhomogeneities. In this case, the local increase in hardness was a consequence of the “structural strengthening” of the matrix by microinhomogeneities of close chemical composition, which created semi-coherent and incoherent boundaries of the Guinier–Preston zone type (Figure 3). The ThixometPRO software (https://thixomet.ru) allowed us to calculate the average diameter of the lacunae, the density of their arrangement, and the percentage of the studied area occupied by these defects. Comparison of the average diameter of the lacunae in two stripes showed that these values are close and amount to ~25 ± 3 μm for the wide stripe and ~22 ± 2 μm for the narrow one. Figure 2 shows that their density on the studied surface differs significantly, and this is confirmed by quantitative calculation: in the wide stripe, the density of lacunae is ~583 pcs/mm^2^, which is 5.6% of the studied area, and in the narrow one, it is ~206 pcs/mm^2^ and occupies 0.95% of the studied area. Outside the wide and narrow stripes, the average diameter of the lacunae is very close and amounts to ~26 ± 1 μm, and their density per unit area is significantly less at ~98 pcs/mm^2^ (see Figure 3).

Figure 4a shows microinhomogeneities with incoherent interfaces located in the region of the stripes (Figure 3). The difference in the structure of the microinhomogeneities and the matrix is so great that during sample preparation the volume of microinhomogeneity is revealed, initially forming cracks marked with red arrows in Figure 4a. The material in the region of the microinhomogeneities can even crumble out of the sample, leaving a gap (cavity), marked with a blue arrow in Figure 4a. In Figure 4b, the interface between such a crumbled microinhomogeneity and the matrix can be distinguished. This image is at the limit of the capabilities of optical microscopy and is therefore not very clear (Figure 4b). The inner surface of the gap is not smooth. It resembles a fine-crystalline surface with structural element sizes less than ~ 1 μm. These structural elements may be localized outlets of needle-like microdomains that appear against the background of a generally monodomain matrix.

In addition to the stripes and microinhomogeneities (Figure 3 and Figure 4), hidden internal defects were also found on the Z-surface, which were recorded almost equally in the bright field, dark field (DF), and DIC mode (Figure 5). Such defects are observed both in the stripe region and outside it, and, apparently, their appearance is in no way related to the causes of the stripes. The nature of these defects is not yet fully understood. In Figure 3, they are indicated by arrows: the red arrows indicate defects hidden in the sample volume, and the yellow arrow indicates a defect that has emerged on the Z-surface of the sample. The DIC mode does not provide additional information to the BF and DF modes (Figure 5a–c). The defect that has an outlet on the surface and its internal structure are shown in Figure 5d,e. Unfortunately, the resolving power of optical microscopy does not allow increasing the information content of the image. At the same time, the image quality is sufficient to see the difference in the boundary surface of the matrix of a defect of this type and microinhomogeneity (Figure 4b and Figure 5d).

The non-polar X- (9 × 8 mm^2^) and Y-faces (7 × 8 mm^2^) of the sample were also examined (Figure 5 and Figure 6). Macroscopic bands similar to those found on the Z-face of the sample (Figure 3) were not detected on the non-polar faces (Figure 5 and Figure 6).

**Figure 7:**
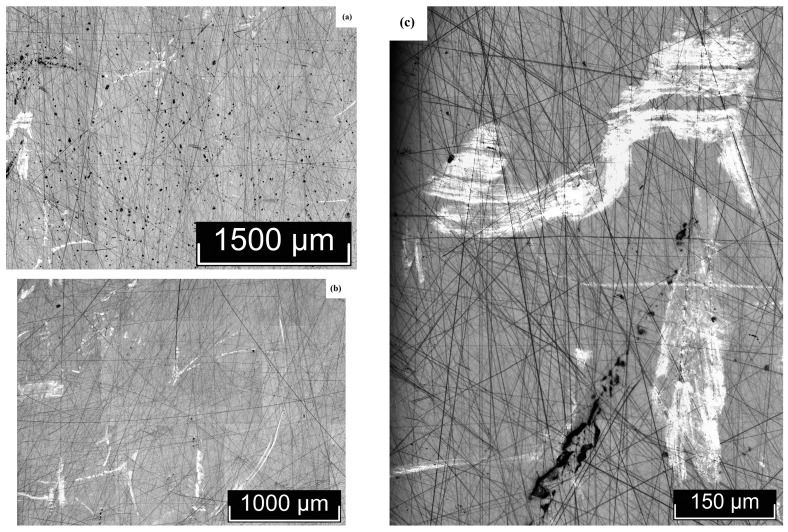
Macro- (**a**,**b**) and microstructures (**c**) of the Y-face of a LiTaO_3_:Cr:Nd crystal sample measuring 7 × 8 mm^2^, studied in the BF mode.

At the same time, irregular defects of various shapes, microinhomogeneities, and lacunae were present on the X- and Y-faces of the sample, similar to the Z-face (Figure 4, Figure 5, Figure 6 and Figure 7). In this case, in Figure 6 and Figure 7, all light defects are located inside the crystal and are detected due to its transparency. Black defects in Figure 6 and Figure 7 are the same defects that have an outlet on the surface. Thus, the appearance of such defects, unlike the bands detected on the Z-face of the sample, does not depend on the crystallographic direction and, apparently, is isotropic in this sense and is determined by growth processes.

It can be assumed that the reason for the formation of such defects may be a relatively high concentration of alloying elements in the melt. This greatly increases its viscosity and leads to the formation of both fairly extended defects, tens to hundreds of microns in size (Figure 4a, Figure 5, Figure 6 and Figure 7), caused by the inhomogeneity of convection movement in the melt, and local fluctuations in the concentration of alloying elements, leading to the appearance of microinhomogeneities and, as a consequence, lacunae (Figure 3, Figure 4, Figure 5d, Figure 6b, and Figure 7a,c).

Quantitative analysis of defects on the X- and Y-faces showed that the lacunae size is comparable to or slightly smaller than on the Z-plane. It is ~22 ± 3 μm on the X-boundary and ~20 ± 1 μm on the Y-boundary. The number of highlighting defects per unit area is also significantly lower: ~105 pcs/mm^2^ on the X-boundary, which, by the way, is quite comparable with the highlighting of defects on the Z-boundary outside the strip (~98 pcs/mm^2^) and ~29 pcs/mm^2^ on the Y-boundary, which is significantly lower than on the Z-boundary. Accordingly, the area occupied by defects is also significantly lower: ~1.43% (X-face) and ~0.5% (Y-face). Based on the transmitted digital data, it can be concluded that possible defects that arise after the emergence in the form of a gap are anisotropic in nature, that is, they depend on the crystallographic direction.

### 3.3. The Conoscopic Patterns of Studied Crystals

**Figure 8:**
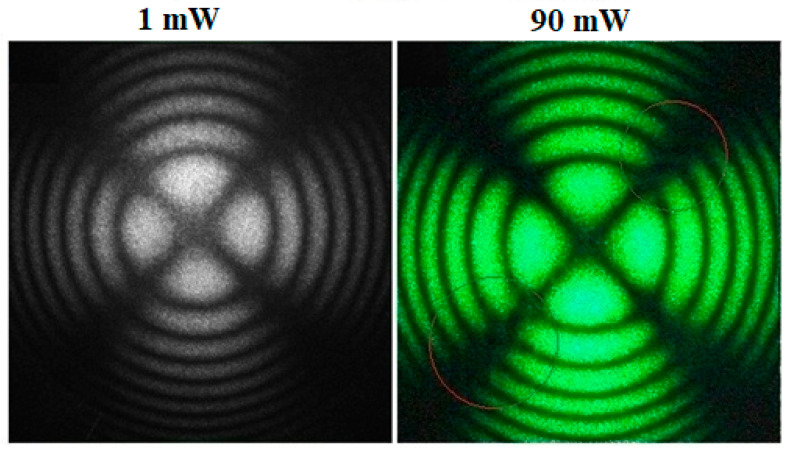
Conoscopic patterns of the LT-8 single crystal at laser radiation powers of 1 and 90 mW. The laser radiation is directed along the polar Z-axis of the crystal.

The conoscopic patterns of all six single crystals studied were identical. Figure 8 shows, as an example, the conoscopic pattern of the LT-8 crystal, which is characterized by the highest values of the concentration of the alloying elements Cr^3+^ and Nd^3+^ in the series of crystals studied and, accordingly, the lowest structural perfection.

The laser conoscopy data indicate that, despite the relatively high concentration of alloying elements Cr^3+^ (0.2 wt%) and Nd^3+^ (0.45 wt%) in the crystal, its conoscopic pattern corresponds to the pattern of a uniaxial crystal with a high degree of structural perfection. Figure 8 clearly shows a completely undistorted “Maltese cross” against the background of isochromes in the form of regular concentric rings. It is also important to note that for all six LiTaO_3_:Cr:Nd crystals studied in the work, the conoscopic pattern of the crystal is more perfect at a laser power of 90 mW than at a laser power of 1 mW (Figure 8). This fact is due to the effect of “healing” defects in the photorefractive LiTaO_3_:Cr:Nd crystal by laser radiation at 90 mW.

In the process of illuminating the analyzed crystal with laser radiation, two competing processes take place. On the one hand, some of the photoelectrons are captured by deep traps and, consequently, uncompensated internal electric fields become distorted by the optical indicatrix and make the conoscopic pattern less “perfect”. On the other hand, with an increase in the power of laser magnification, the efficiency of the competing process increases—radiative recombination of photoexciting carriers without their capture at deep depths. Consequently, uncompensated main electric fields are reduced, distorting the optical indicatrix and leading in a single-domain piezoelectric crystal, among other things, to the occurrence of a piezo-optic effect, continuing in the expansion of optical anisotropy under the action of local mechanical stresses caused by uncompensated electric fields. Thus, an increase in the width of laser radiation leads to a partial annealing of charged defects and, accordingly, to achieving perfection of the conoscopic pattern. In this case, the effect of annealing (“healing”) of some types of charged defects in optical crystals is observed when exposed to laser radiation, which is known in the literature [61,62,63].

### 3.4. Transmission Spectra of Studied Crystals

**Figure 9:**
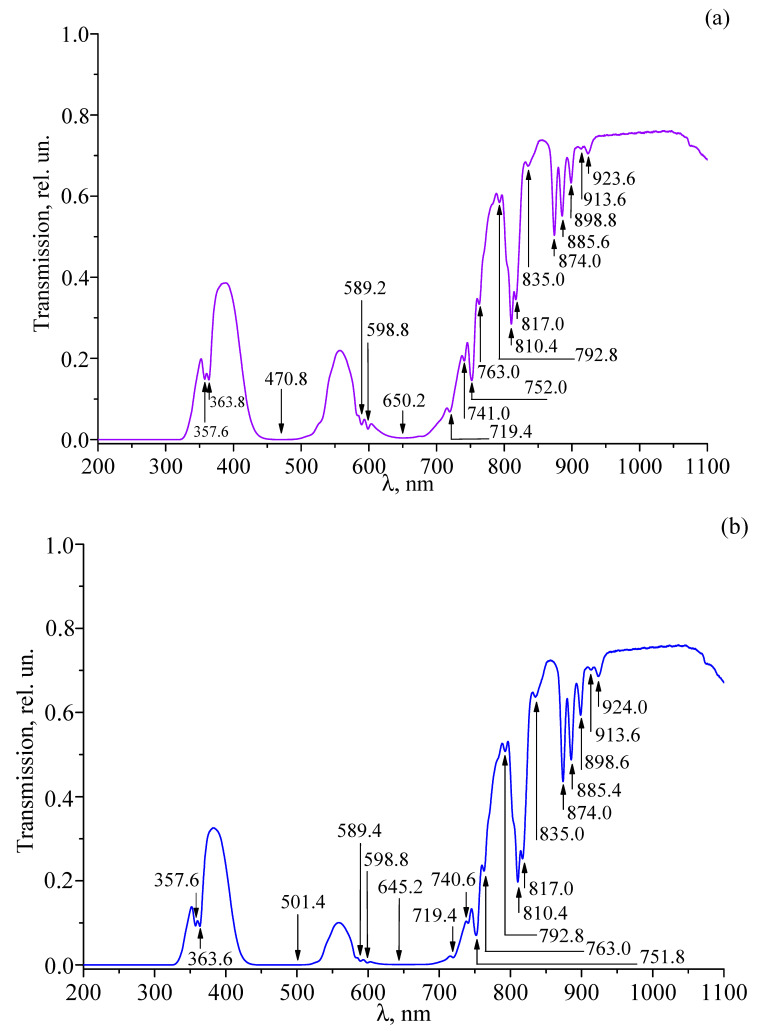

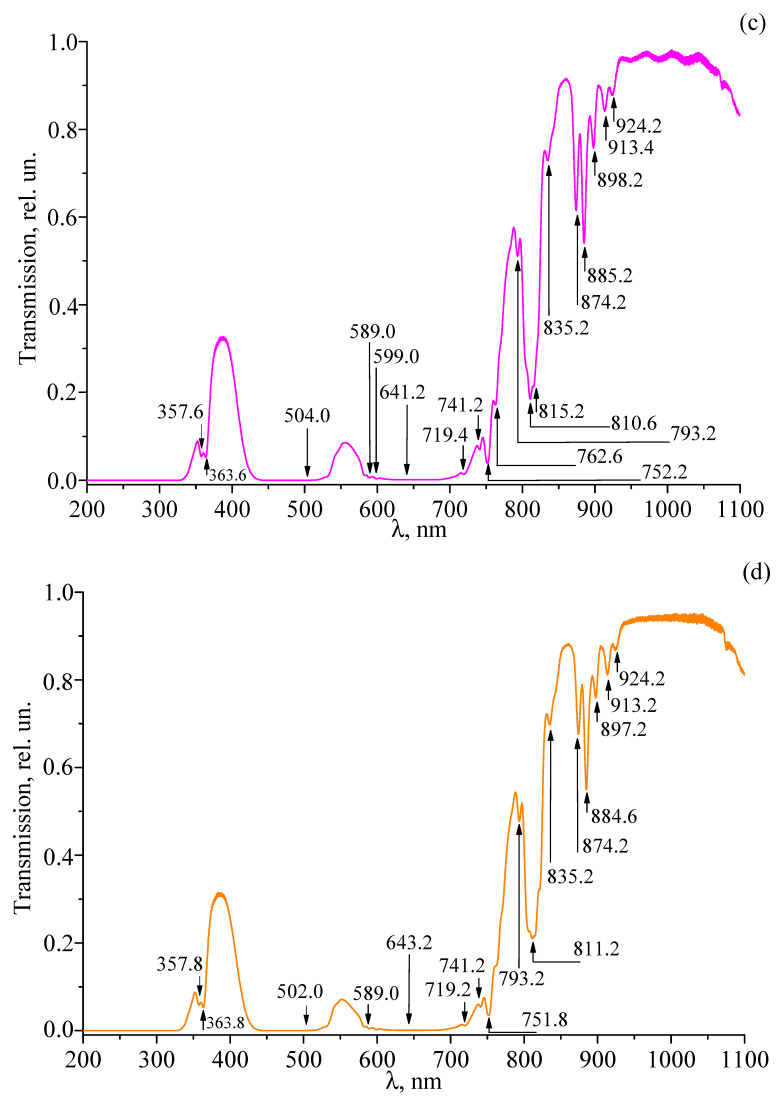

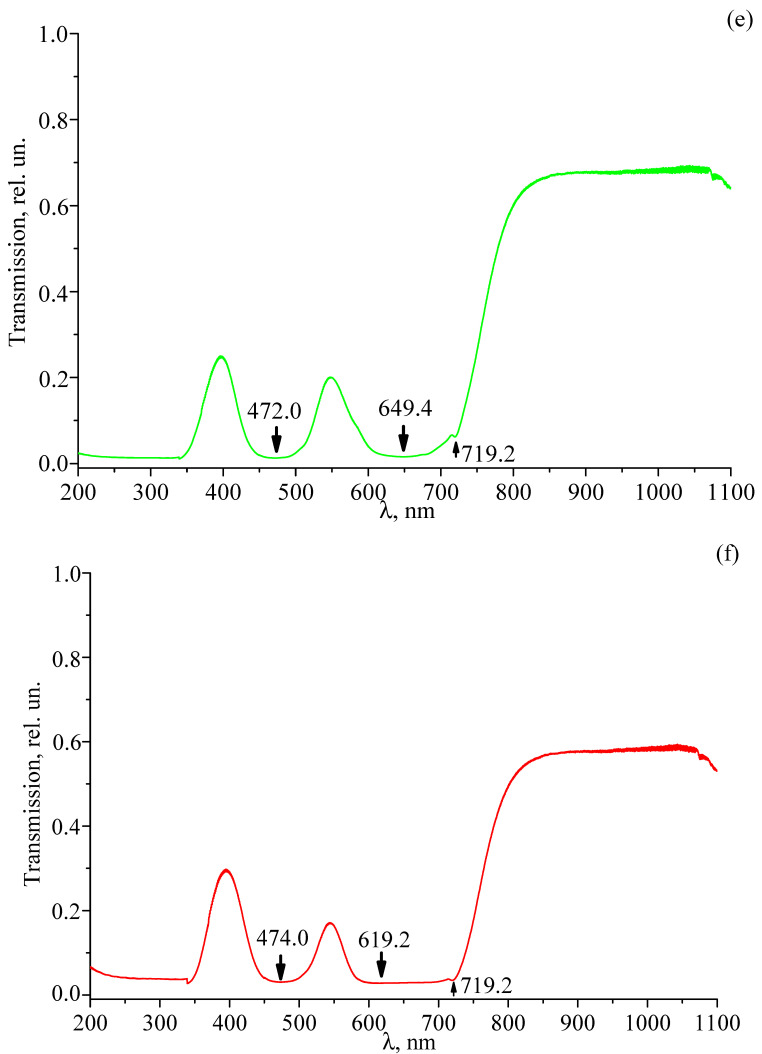
Transmission spectra of crystals: LT-3 (**a**), LT-4 (**b**), LT-5 (**c**), LT-6 (**d**), LT-7 (**e**), and LT-8 (**f**).

Figure 9a–f shows the transmission spectra of the studied double-doped LiTaO_3_:Cr^3+^:Nd^3+^ single crystals recorded in the wavelength range of 200–1100 nm. The experimentally observed absorption wavelengths and their corresponding assignments are summarized in Table 3. It is evident that all the samples are opaque below 325 nm. It is also evident that changing the concentration of Cr^3+^ and Nd^3+^ ions in the crystal significantly changes the shape of the transmission spectra. At concentrations of Cr^3+^ ions of 0.09 wt% or less and Nd^3+^ ions of 0.28 wt% or less, the spectra contain a number of narrow bands corresponding to absorption in Nd^3+^ ions, two broad absorption bands corresponding to absorption in Cr^3+^ ions, and a fundamental absorption band of the lithium tantalate matrix (Figure 9a–d). At concentrations of Cr^3+^ ions of 0.11 wt% or more and Nd^3+^ ions of 0.41 wt% or more, the narrow absorption lines of Nd^3+^ ions disappear from the spectra (Figure 9e,f). Table 3 shows that the majority of the observed absorption lines are related to electronic transitions in Nd^3+^ ions, while only three lines correspond to transitions in Cr^3+^ ions. For some recorded absorption lines, the assignment is unclear. This may be due to the presence of uncontrolled (trace) amounts of impurities of other metals in the crystals, the concentration of which is small and amounts to <10^−4^ wt% [1].

It is interesting to note that the concentration behavior of the absorption lines in the optical spectrum of LiTaO_3_:Cr^3+^:Nd^3+^ crystals correlates well with the concentration behavior of the lines in the spectrum of LiTaO_3_:Mg^2+^:Nd^3+^ crystals studied in [18,19,20,21,39]. Namely, at low concentrations of Nd^3+^ and Mg^2+^ ions, a number of narrow lines corresponding to absorption in Nd^3+^ ions and two broad absorption bands corresponding to absorption in Mg^2+^ ions are observed in the spectrum of LiTaO_3_:Mg^2+^:Nd^3+^ crystal, as well as in the spectrum of LiTaO_3_:Cr^3+^:Nd^3+^ crystal. At high concentrations of Nd^3+^ and Mg^2+^ ions, the narrow absorption lines of Nd^3+^ ions disappear from the spectra.

The fact that the narrow absorption lines of Nd^3+^ ions disappear from the transmission spectrum at high concentrations of Mg^2+^, Cr^3+^, and Nd^3+^ ions can be explained by a significant increase in the structural disorder in the cation sublattice of double-doped LiTaO_3_:Mg^2+^:Nd^3+^ and LiTaO_3_:Cr^3+^:Nd^3+^ crystals at high concentrations of doping ions. Indeed, in the structure of LiNbO_3_ and LiTaO_3_ crystals, the size of the lithium octahedron O_6_ is larger than the sizes of the niobium and vacant O_6_ octahedra [1]. Therefore, at low doping levels, when the concentration of doping ions in the LiTaO_3_ crystal is low, it is energetically favorable for the doping ions Mg^2+^, Cr^3+^, and Nd^3+^ to primarily occupy the lithium octahedra of the structure, displacing the point defects of the Nb_Li_ cation sublattice (Nb^5+^ ions located in the position of the Li^+^ ions of the ideal structure). In this case, the stoichiometry of the crystal increases, and the ordering of the structural units of the cation sublattice along the polar axis increases due to a decrease in the number of Nb_Li_ defects and, accordingly, a decrease in the number of vacant O_6_ octahedra. Such an increase in the ordering of the structural units of the cation sublattice leads to an increase in the resistance of the crystal to optical damage, since the point defects of Nb_Li_ are deep electron traps that enhance the photorefraction effect [1,64,65]. In addition, with the ordering of the structural units of the cation sublattice along the polar axis of the crystal, the shape of the oxygen-octahedral clusters of the MeO_6_ structure (Me–Li, Nb, V vacancy, impurity metal) becomes more perfect. Such an effect of structural ordering was observed according to X-ray diffraction and Raman analysis data in single-doped LiNbO_3_:Zn^2+^ and LiNbO_3_:Mg^2+^ crystals, as well as in double-doped LiNbO_3_:Zn^2+^:Mg^2+^ crystals [14,15]. In this case, in the Raman spectrum of doped crystals in a certain concentration region of zinc and magnesium, a decrease in the line widths was observed, which indicates a more perfect crystal structure [66,67]. The maximum ordering of the structural units of the LiNbO_3_:Zn crystal was observed at 0.05–0.94 mol% ZnO [67]. Thus, at low concentrations of the doping ions Mg^2+^, Cr^3+^, and Nd^3+^, the structure of the double-doped LiTaO_3_ crystal as a whole becomes more perfect and less defective, which leads to an improvement in the spectral characteristics of the crystal. At low concentrations of the doping ions in the LiTaO_3_:Cr^3+^:Nd^3+^ crystal, the existence of three nonequivalent neodymium centers is observed for radiative transition in the cation sublattice and two nonequivalent centers for the radiative transition (Table 3).

With increasing concentration, the doping ions Mg^2+^, Cr^3+^, and Nd^3+^ in the LiTaO_3_ crystal structure begin to displace not only the Nb_Li_ point defects from their positions, but also other cations and occupy the vacant O_6_ octahedra. In this case, incorporation of a doping ion with a certain charge into the neutral vacant oxygen octahedron O_6_, along with a decrease in Li^+^ vacancies (V_Li_ defect), leads to an additional increase in the defectiveness of the cation sublattice and a statistical distribution of the doping ions (not included in the lithium octahedron) over the oxygen octahedra of the structure. These factors lead to a broadening of the spectral lines. With strong disordering of the cation sublattice, due to significant line broadening, the narrow lines of the Nd^3+^ ion merge into wide bands, the intensity of the narrow lines decreases, and they disappear from the optical spectrum. Accordingly, strong disordering of the cation sublattice leads to deformation of oxygen-octahedral clusters MeO_6_, responsible for the nonlinear optical and ferroelectric properties of the crystal. At the same time, the resistance of the crystal to damage by optical radiation also decreases.

The strongest increase in the disorder of the cation sublattice occurs at concentration thresholds, when the state of crystal defectiveness changes abruptly due to an abrupt change in the mechanism of entry of doping cations into the crystal structure [63]. However, at the concentration threshold, the space symmetry group of the unit cell of the crystal and the number of formula units in it do not change, but the parameters of the unit cell change insignificantly abruptly [66]. According to the literature, we have not found information on the presence of concentration thresholds in LiTaO_3_ crystals doped with various metals. However, concentration thresholds are characteristic of doped LiNbO_3_ crystals isomorphic to the LiTaO_3_ crystal [1,14,66]. It is possible that the absence of concentration thresholds in doped LiTaO_3_ crystals is due to a higher disordering of the LiTaO_3_ crystal structure compared to the LiNbO_3_ crystal structure. For this reason, the concentration thresholds can be initially “smeared” by the high structural disorder of the LiTaO_3_ crystal. The LiTa_y_Nb_1-y_O_3_ system does not form single-crystal solid solutions in the entire range of y [1]. According to the data of [1], the transformation of the LiNbO_3_ crystal structure into the LiTaO_3_ crystal structure in the LiTa_y_Nb_1-y_O_3_ solid ceramic solution system occurs through a certain intermediate structure (existing in the region of average y values), which differs slightly from the structures of LiNbO_3_ and LiTaO_3_ crystals. The oxygen octahedra O_6_ of this intermediate structure are noticeably distorted in comparison with the octahedra O_6_ in the structures of LiNbO_3_ and LiTaO_3_ crystals, and their symmetry is lowered. The distortion of the oxygen octahedra O_6_ in the region of average values of y is caused by the unequal value of the Nb-O and Ta-O bonds in the oxygen clusters NbO_6_ and TaO_6_. In this case, the entry of niobium and tantalum ions into “foreign” positions and the formation of corresponding point defects distorting the oxygen-octahedral clusters MeO_6_ are inevitable.

Previously [68], an attempt was made to find the optimal concentration of chromium ions in a lithium niobate crystal. The authors analyzed the transmission spectra of LiNbO_3_:Cr^3+^(0.1, 0.25, and 0.5 mol%) crystals. It turned out that for chromium concentrations of 0.25 and 0.5 mol%, all three absorption bands, ^4^A_2_→^4^T_1_, ^4^A_2_→^4^T_2_, and ^4^A_2_→^2^E, appear, while at a concentration of 0.1 mol%, only two bands, ^4^A_2_→^4^T_2_ and ^4^A_2_→^2^E, appear. On the other hand, with an increase in the chromium ion concentration from 0.1 to 0.5 mol%, the transmittance of the crystal in the range of 900–1500 nm drops by 10–15%. As a result, according to the authors of the article [68], for the LiNbO_3_:Cr^3+^ crystal, the optimal concentration of chromium ions is 0.25 mol% in the transmission geometry and 0.5 mol% for the reflection geometry. For the crystal grown by the Bridgman method, the results are close [69].

According to the referenced articles, the optimal concentration of chromium and neodymium in a LiTaO_3_ crystal is achieved by meeting three criteria: ensuring all characteristic absorption lines are present, maintaining at least 50% light transmission in the 900–1500 nm range, and minimizing structural defects.

### 3.5. Raman Spectra of Studied Crystals

**Figure 10:**
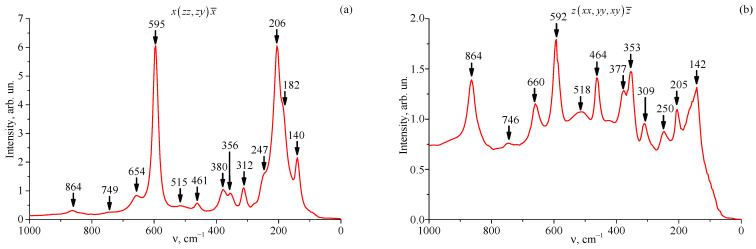

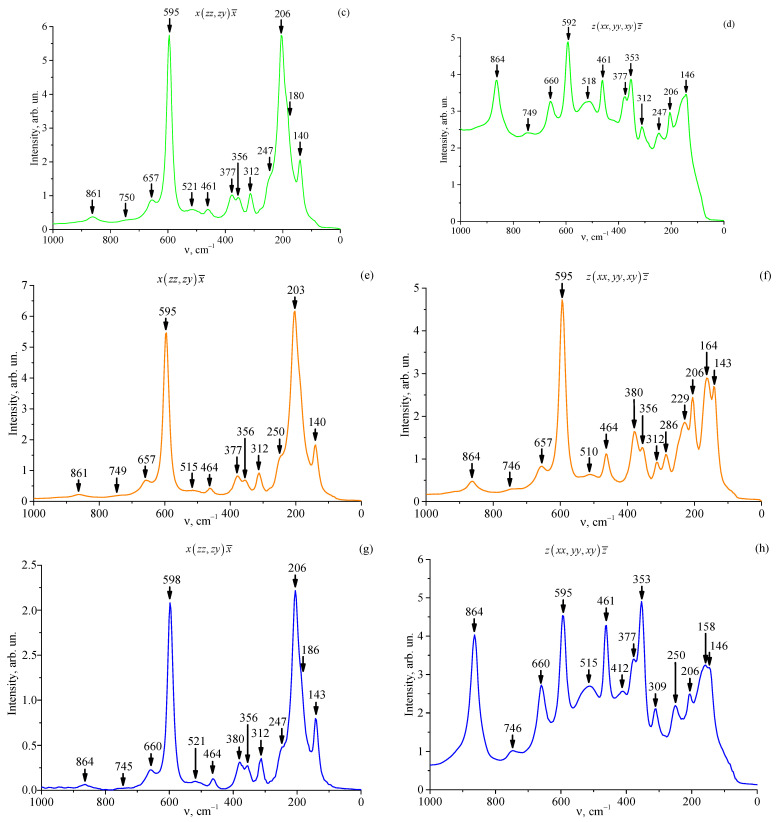

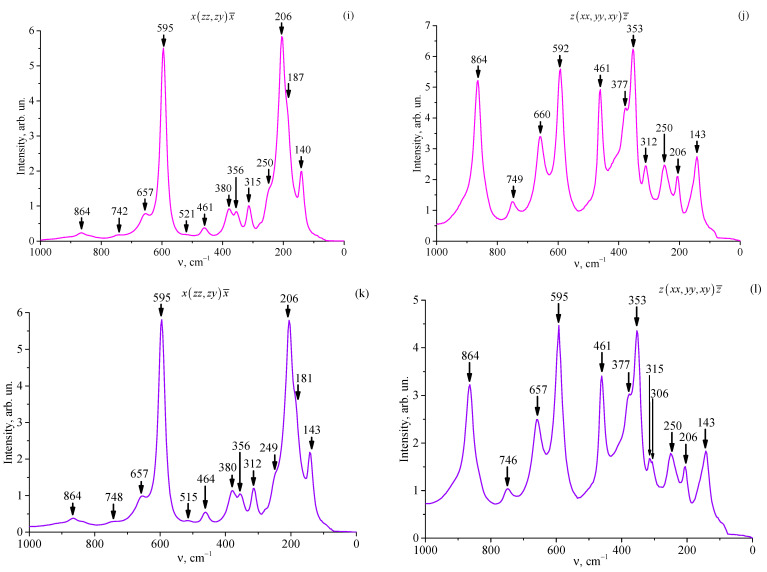
Raman spectra of crystals LT-3 (**a**,**b**); LT-4 (**c**,**d**); LT-5 (**e**,**f**); LT-6 (**g**,**h**); LT-7 (**i**,**j**); LT-8 (**k**,**l**).

The increase in the structural disorder of LiTaO_3_:Cr^3+^:Nd^3+^ crystals with increasing concentration of Cr^3+^ and Nd^3+^ ions is also confirmed by Raman spectroscopy data. Figure 10 shows the recorded Raman spectra of the studied crystals, recorded in the region of fundamental vibrations of the crystal lattice (100–1000 cm^−1^).

Table 4 shows the frequency values of the experimentally observed Raman spectrum lines, as well as their assignment, performed using the literature data [70,71,72] for a nominally pure LiTaO_3_ crystal.

As can be seen, in the $z\left(\mathrm{xx},\mathrm{yy},\mathrm{xy}\right)\;\bar{z}$ scattering geometry, the luminescent background has a higher intensity. It should be especially noted that for the LT-5 crystal, there is a “violation” of this scattering geometry—the intensity of the Raman line with a frequency of 864 cm^−1^ is less than that of the other Raman lines.

From Figure 10 and Table 4, it is evident that in the Raman spectrum, in addition to the lines allowed by the selection rules for the space group of the $C_{3V}^{6}$ (R3c) unit cell with two formula units in it, a number of additional low-intensity lines are observed that are not provided for by the selection rules, which confirms the fact of a more disordered structure of LiTaO_3_:Cr^3+^:Nd^3+^ crystals compared to the structure of a nominally pure LiTaO_3_ crystal. In particular, the lines with frequencies of 158 and 164 cm^−1^, not observed in [70,71] in the Raman spectrum of a nominally pure LiTaO_3_ crystal, were assigned to the 2E(LO)-mode in [73]. In [74], a line with a frequency of 228 cm^−1^ was discovered in the Raman spectrum of a lightly doped LiTaO_3_:Nd^3+^(0.1 wt%) crystal in the y(zy)x scattering geometry. The authors attribute the appearance of a specific Raman line at 286 cm^−1^ to the photorefraction effect. This effect is known to make spectral lines appear even when they are forbidden by selection rules for a given scattering geometry. Furthermore, calculations from reference [70] identify this specific line as the fully symmetric 2A_1_(z)TO-mode. The highest-frequency line, 746–749 cm^−1^, can be attributed to the A_2_ mode forbidden by the selection rules [72]. The assignment of the remaining recorded Raman spectrum lines with frequencies of 306, 412, and 510–521 cm^−1^ remains unclear.

### 3.6. FTIR Absorption Spectra of Studied Crystals

The structure of nominally pure and doped LiTaO_3_ crystals can be considered as a sequence of oxygen-octahedral clusters МеО_6_ (Ме- Li^+^, Ta^5+^, dopant, vacant octahedron V), connected by common faces and edges along the polar axis [1]. The МеО_6_ clusters are filled by one-third with Ta^5+^ ions, by one-third with Li^+^ ions, while one-third of the clusters remain vacant. This structural feature allows doping of the LiTaO_3_ crystal with a wide range of metals, including rare earth and transition metals [5,6]. When an alloying metal (Me) is introduced as a defect into the LiTaO_3_ crystal structure, it can occupy one of three locations: replacing Li^+^ ions, replacing Ta^5+^ ions, or settling into a vacant octahedron (O_6_). The specific location depends on the crystal’s composition and growth method, and the resulting point defects (Me_Li_, Me_Ta_, and Me_V_) act as deep electron traps that control the magnitude of the photorefraction effect. At the same time, due to the preservation of the electroneutrality of the crystal, smaller electron traps are formed—point defects V_Me_, etc. Defects in the form of small and deep electron traps also play a significant role in the formation of the optical characteristics of LiTaO_3_ crystals. It should be noted that due to the large ionic radii compared to the radii of Li^+^ and Ta^5+^ ions (68.0, 68.0 pm), it is unlikely that rare earth ions Nd^3+^ (99.5 pm) will be located in the vacant octahedron of the ideal structure, the volume of which is less than the volume of the occupied octahedron [1].

**Figure 11:**
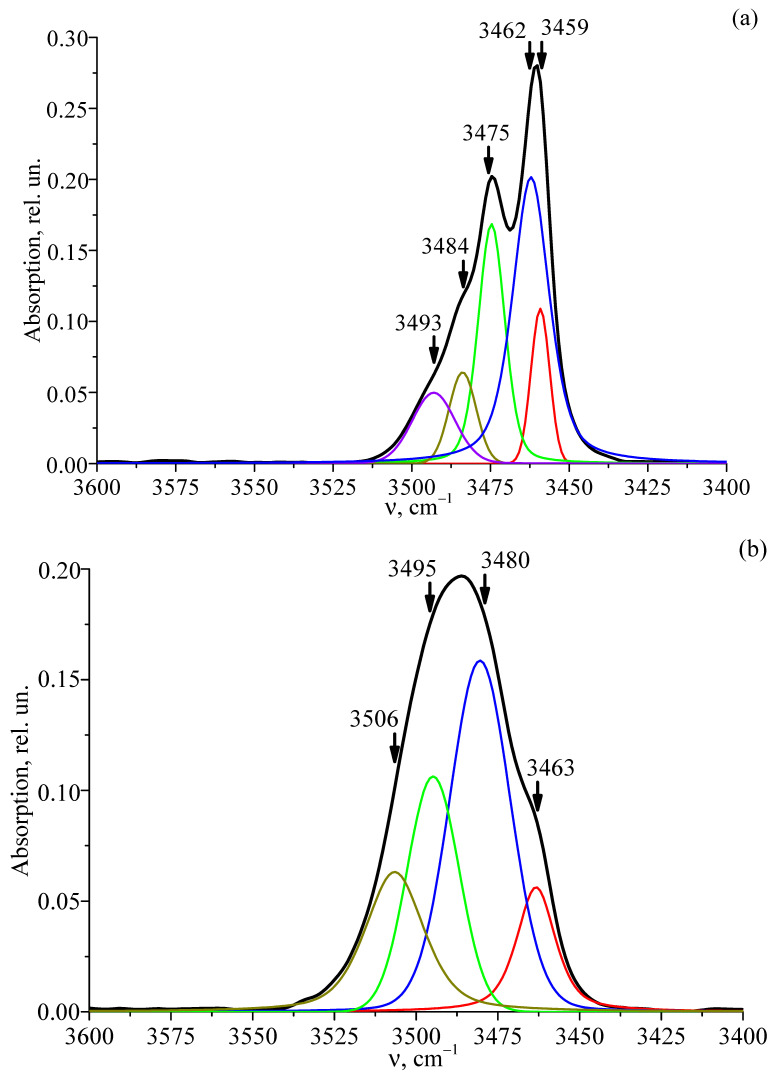

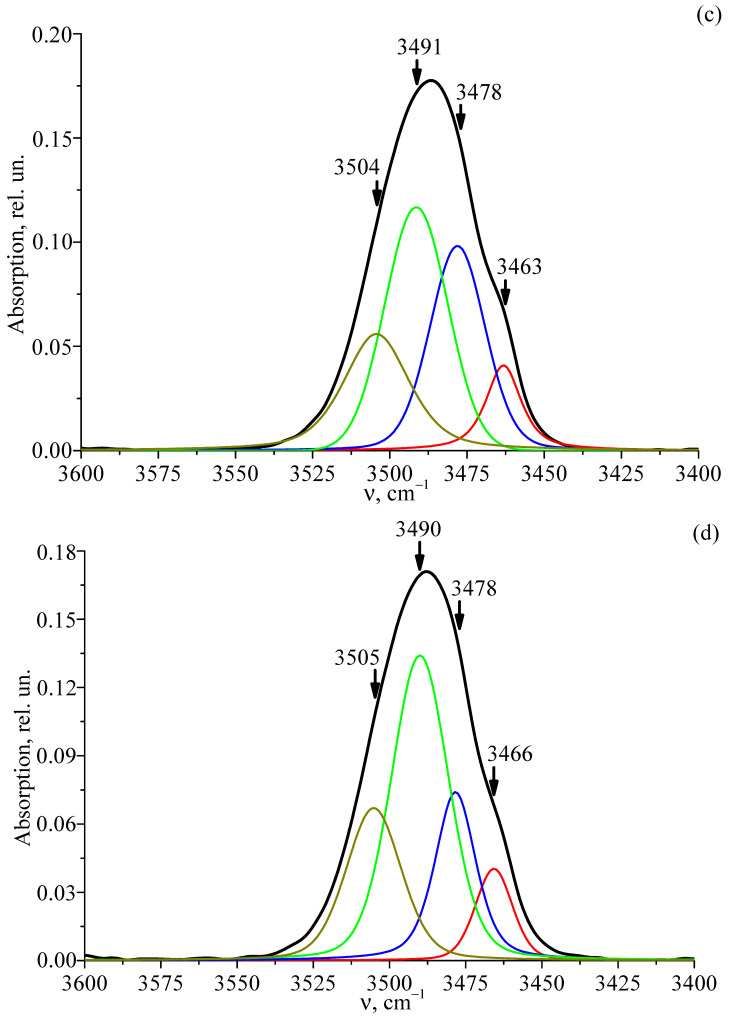

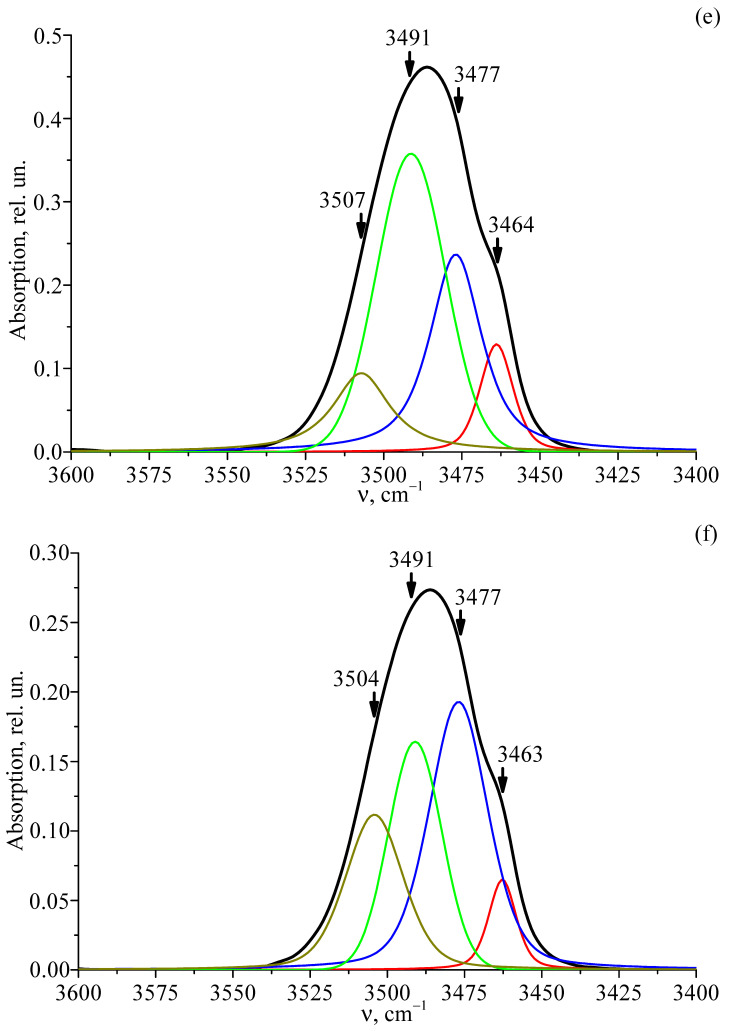

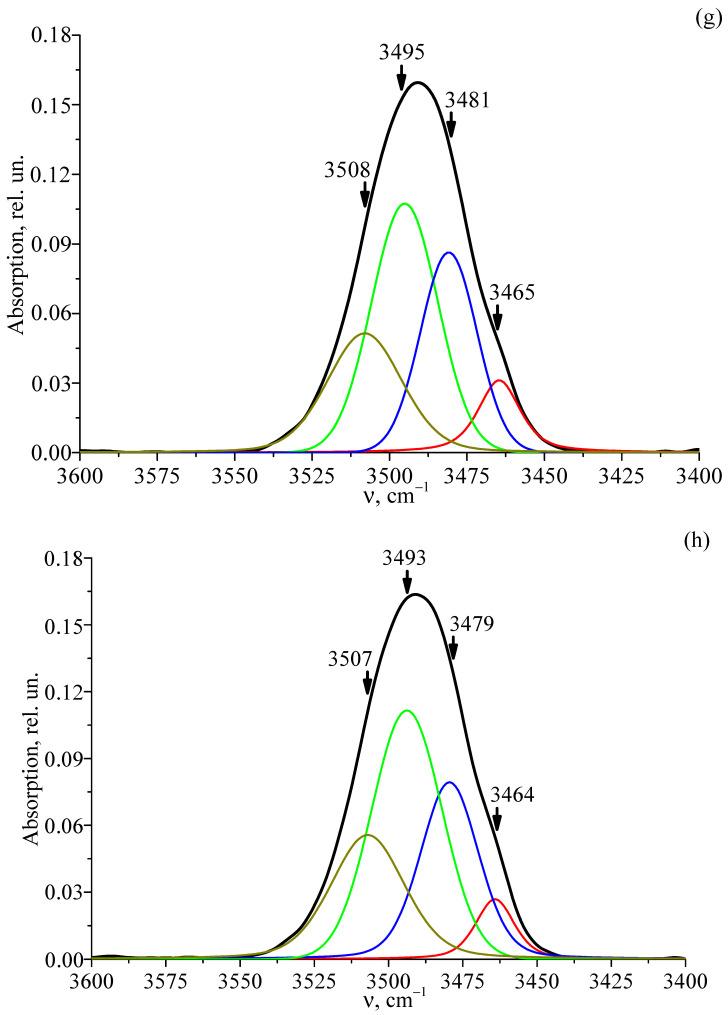
FTIR absorption spectra in the region of stretching vibrations of OH^−^ groups of single crystals: LT-1 (**a**), LT-2 (**b**), LT-3 (**c**), LT-4 (**d**), LT-5 (**e**), LT-6 (**f**), LT-7 (**g**), LT-8 (**h**).

Figure 11 shows the FTIR absorption spectra of the LT-1 crystal, the single-doped LT-2 crystal, and the double-doped LT-3, LT-4, LT-5, LT-6, LT-7, and LT-8 crystals recorded in the frequency range of 3400–3600 cm^−1^, where the stretching vibrations of the OH^−^ groups appear. Table 5 shows the values of the spectral line parameters: frequency, width, and intensity. By analogy with the extensively studied LiNbO_3_ [40,41,42,43], two sets of spectral lines observed in all crystals can be attributed to different causes. The lines in the 3463–3465 cm^−1^ range result from changes in the crystal’s stoichiometry (R value), whereas those in the 3486–3490 cm^−1^ range are due to the formation of complex OH^−^ defects involving the heavy Ta^5+^ ion (180.95 amu) occupying Li^+^ sites.

It is evident from Figure 11 that the low-intensity line with a frequency of ≈3504 cm^−1^ is present only in the spectra of doped LiTaO_3_ crystals. The appearance of this line in the spectrum is attributed to point defects of (Nd^2+^)_Li_ and to associated complex defects involving OH^−^ groups. In these defects, the lighter Nd^3+^ ion (144.24 amu) replaces the Li^+^ ion, in contrast to the heavier Ta^5+^ ion (180.95 amu), resulting in the formation of the (Nd^3+^_Li_)-OH defect. The presence of complex defects such as (Ta^4+^_Li_)-OH and (Nd^2+^_Li_)-OH leads to significant deformation of the corresponding oxygen-octahedral clusters (MeO_6_). This deformation, which is caused by changes in the O–O and Me–O bond lengths, is more pronounced when Nd^3+^ (ionic radius 99.5 pm) is involved compared to Ta^5+^ (68.0 pm). Consequently, the oxygen-octahedral cluster is more significantly distorted, resulting in a stronger broadening of the FTIR spectral lines associated with the (Nd^2+^_Li_)-OH defects (see Figure 11, Table 5). At the same time, the presence of point defects Cr^2+^_Li_ and the associated complex defects (Cr^2+^_Li_)-OH slightly distorts the oxygen-octahedral clusters МеО_6_, since the ionic radii of Cr^3+^, Ta^5+^, and Li^+^ are close (61.5, 68.0, 68.0 pm). Therefore, the line corresponding to the stretching vibrations of hydrogen atoms in the (Cr^2+^_Li_)-OH defect in the vibrational spectrum has practically the same frequency as in the (Ta^4+^_Li_)-OH and (Nd^2+^_Li_)-OH defects and is not observed separately in the spectrum. Other complex defects involving OH^−^ groups, the concentration of which is small, are also not manifested in the spectrum. Accordingly, the intensity of the lines corresponding to the stretching vibrations of hydrogen atoms in such an OH^−^ group will also be small.

According to the data of work [75], the doping Cr^3+^ cations occupy the main positions of the Ta^5+^ ions in the LiTaO_3_ crystal. At the same time, in later works [9,10], it was concluded that the Cr^3+^ ions occupy both the positions of the Ta^5+^ ion and the main positions of the Li^+^ ion of the ideal structure. This arrangement of point defects leads to the formation of a complex defect pair in the form of adjacent point defects Cr^2−^_Ta_ and Cr^2+^_Li_. A hydrogen atom is attracted to this pair of defects, forming a complex defect (Cr^2+^_Li_)-OH-(Cr^2−^_Ta_). Nd^3+^ ions, which have a larger ionic radius than Cr^3+^ ions (99.5 and 61.5 pm, respectively), and located in the lithium position of the ideal structure, form a point defect center Nd^2+^_Li_.

Both modes of Cr^3+^ ion localization in the crystal structure resulted in a slight increase in the quasi-elastic constant of the hydrogen bond. This, in turn, produced a small shift in the hydrogen atoms’ stretching vibration frequency toward higher values and led to the emergence of an absorption line at 3490 cm^−1^ in the spectra of the LT-7 and LT-8 crystals (see Figure 11). Increasing the concentration of Nd^3+^ and Cr^3+^ doping ions enhances the formation of self-compensating defect pairs (Nd^2+^_Li_–Nd^2+^_Ta_ and Cr^2+^_Li_–Cr^2+^_Ta_). These defect pairs exert a stronger attractive force on the hydrogen atoms bound to oxygen, leading to the creation of complex defects: (Nd^2+^_Ta_)-OH, (Cr^2+^_Ta_)-OH, (Nd^2+^_Li_)-OH-(Nd^2+^_Ta_), and (Cr^2+^_Li_)-OH-(Cr^2+^_Ta_). The formation of these complex defects noticeably distorts the configuration of the oxygen-octahedral clusters (MeO_6_), which is reflected in the broadening of the spectral lines associated with the stretching vibrations of hydrogen atoms in these defects (see Figure 11, Table 5).

Thus, the greatest changes in the parameters of the FTIR absorption spectra of the studied crystals (frequency, width, and intensity) are observed in the frequency range of 3450–3490 cm^−1^, i.e., they are associated with a change in the stoichiometry (R value) of LiTaO_3_ crystals during doping. At the same time, with a change in the concentration of the doping elements Nd^3+^ and Cr^3+^, it is possible for the defective structure of the crystal to pass through concentration thresholds.

The concentration of point defects and related complex defects involving OH^−^ groups determines the volume concentration of OH^−^ groups in the LiTaO_3_ crystal. Based on the FTIR absorption spectra, using the Klauer method [76,77], we determined the volume concentration of OH^−^ groups in the studied crystals. Table 5 shows that in LiTaO_3_:Cr^3+^:Nd^3+^ crystals, the change in the concentration of OH^−^-groups with a change in the crystal composition is non-monotonic. This may be due to the presence of concentration thresholds formed as a result of an abrupt increase in the number of defect centers of different types, both point and complex. It can be assumed that the first concentration threshold in the LT-5 crystal is formed as a result of the simultaneous formation of two types of complex defects: (Cr^2+^_Li_)-OH-(Cr^3−^_Ta_) and (V^−^_Li_)-OH. In this case, for this crystal, the maximum concentration of OH^−^ groups in the series of crystals studied is observed (Table 5) and the maximum changes in the FTIR absorption spectrum are observed (Figure 11e).

## 4. Conclusions

It is shown that the X-ray patterns of all six investigated LiTaO_3_:Cr:Nd crystals are identical and correspond to the highly perfect structure of the LiTaO_3_ crystal, the unit cell of which is characterized by the space group $C_{3V}^{6}$ (R3c). Reflections corresponding to impurity phases are not detected. The micro- and macrostructures of single crystals are investigated by optical microscopy. The presence of defects of various shapes, microinhomogeneities, and lacunae is detected.

It was found that at Cr^3+^ ion concentrations of 0.09 wt% or less and Nd^3+^ ion concentrations of 0.28 wt% or less, the optical transmission spectra of LiTaO_3_:Cr^3+^:Nd^3+^ crystals contain a number of narrow lines corresponding to electron transitions in Nd^3+^ ions, as well as two broad absorption bands corresponding to electron transitions in Cr^3+^ ions and a fundamental absorption band of the lithium tantalate matrix. At Cr^3+^ ion concentrations of 0.11 wt% or more and Nd^3+^ ion concentrations of 0.41 wt% or more (due to an increase in the structural disordering of the crystal), the narrow absorption lines of Nd^3+^ ions disappear from the transmission spectra.

The fact of increasing structural disordering of LiTaO_3_:Cr^3+^:Nd^3+^ crystals at concentrations of Cr^3+^ ions of 0.11 wt% and higher and Nd^3+^ ions of 0.41 wt% and higher is also confirmed by Raman spectroscopy data. Thus, at concentrations of doping ions Cr^3+^ and Nd^3+^ less than 0.09 wt%, the structure of the double-doped LiTaO_3_ crystal as a whole becomes more perfect and less defective, which leads to an improvement in the spectral characteristics of the crystal. At concentrations of doping ions Cr^3+^ and Nd^3+^ less than 0.09 wt% in the LiTaO_3_:Cr^3+^:Nd^3+^ crystal, the existence of three nonequivalent neodymium centers is observed for the radiative transition in the cation sublattice, and two nonequivalent centers for the radiative transition.

Based on the FTIR absorption spectra in the region of frequencies of stretching vibrations of hydrogen atoms belonging to OH^−^ groups of LiTaO_3_, LiTaO_3_:Cr^3+^, and LiTaO_3_:Cr^3+^:Nd^3+^ crystals, it was established that in the crystals, along with point defects Ta^5+^_Li_, Nd^3+^_Li_, Cr^3+^_Li_, V_Li_, etc., there are numerous complex defects—(Ta^5+^_Li_)-OH, (Nd^2+^_Li_)-OH, (Cr^2+^_Li_)-OH, (V^−^_Li_)-OH, etc., caused by the presence of hydrogen bonds. In this case, in the FTIR absorption spectrum, lines with frequencies in the range of 3463–3465 and 3486–3490 cm^−1^ are caused, respectively, by a change in the crystal stoichiometry (R value) and the formation of complex defects (V^−^_Li_)-OH and (Ta^5+^_Li_)-OH. It was found that a low-intensity line with a frequency of ≈3504 cm^−1^ is present only in the spectrum of doped lithium tantalate crystals. Its appearance in the spectrum is attributed to complex defects (Nd^2+^_Li_)-OH. These defects cause noticeable deformation of the oxygen-octahedral clusters (MeO_6_, which include Me-Li^+^, Ta^5+^, the alloying element, and a vacant octahedron V) by altering the O–O and Me–O bond lengths. The deformation is primarily due to the doping cation Nd, which has a larger ionic radius (99.5 pm) compared to Ta^5+^ (68.0 pm). It is shown that the appearance of point defects Cr^2+^_Li_ slightly distorts the oxygen-octahedral clusters МеО_6_, since the ionic radii of Cr^3+^, Ta^5+^, and Li^+^ are close. The Klauer method was employed to measure the volumetric concentration of OH^−^ groups in the LiTaO_3_ crystals. The highest concentration was observed in the LiTaO_3_:Cr^3+^(0.09 wt%):Nd^3+^(0.25 wt%) crystal. This peak is attributed not only to the presence of (Nd^2+^_Li_)-OH defects but also to the fact that this crystal exhibits the greatest number of two additional complex defects: (Cr^2+^_Ta_)-OH-(Cr^2+^_Li_) and (V^−^_Li_)-OH. The obtained results allow us to conclude that there are threshold effects in the LiTaO_3_:Cr^3+^(0.09):Nd^3+^(0.25 wt%) crystal. They arise due to the simultaneous occurrence of two types of complex defects in the structure: (Cr^3+^_Li_)-OH-(Cr^3−^_Ta_) and (V^−^_Li_)-OH.

## Figures and Tables

**Table 1:** Concentration of dopants in LiTaO_3_:Cr:Nd crystals.

Crystal	Crystal Composition	[Cr], wt%	[Nd], wt%
LT-1	LiTaO_3_	-	-
LT-2	LiTaO_3_:Cr	0.005	-
LT-3	LiTaO_3_:Cr:Nd	0.06	0.2
LT-4	LiTaO_3_:Cr:Nd	0.07	0.28
LT-5	LiTaO_3_:Cr:Nd	0.09	0.25
LT-6	LiTaO_3_:Cr:Nd	0.1	0.25
LT-7	LiTaO_3_:Cr:Nd	0.11	0.41
LT-8	LiTaO_3_:Cr:Nd	0.2	0.45

**Table 2:** Impurity composition of the initial batch of LiTaO_3_ and LT-5 crystals.

Admixture	Concentration of Impurities in the Batch LiTaO_3_, wt%	Impurity Content in the Crystal LT-5, wt%
Mn, V, Mg, Sn	<5·10^−4^	<5·10^−4^
Pb, Ni, Co	<1·10^−3^	<1·10^−3^
Mo	<1·10^−3^	<1·10^−3^
Si, Fe	<1·10^−3^	<1·10^−3^
Ti	<1·10^−3^	<1·10^−3^
Al	<5·10^−4^	<5·10^−4^
Zr	<1·10^−2^	<1·10^−2^
Ca	<5·10^−3^	<5·10^−3^
Cu	<5·10^−4^	<5·10^−4^
T_C_, °C	1145.0	1145.0

**Table 3:** Wavelength values (nm) of experimentally observed absorption lines in the transmission spectrum of LiTaO_3_:Cr^3+^:Nd^3+^ crystals and their assignment to the electronic transitions of Cr^3+^ and Nd^3+^ ions.

LT-3	LT-4	LT-5	LT-6	LT-7	LT-8	Transition
357.6	357.6	357.6	357.8			${\mathrm{Nd}}^{3+}{:I}_{9/2}^{4}\to D_{1/2}^{4}$
363.8	363.6	363.6	363.8			${\mathrm{Nd}}^{3+}{:I}_{9/2}^{4}\to D_{3/2}^{4}$
470.8						${\mathrm{Cr}}^{3+}{:A}_{2}^{4}\to T_{1}^{4}$
				472.0		
					474.0	
	501.4					
			502.0			
		504.0				
589.2	589.4	589.0	589.0			${\mathrm{Nd}}^{3+}{:I}_{9/2}^{4}\to G_{5/2}^{4}{+G}_{7/2}^{2}$
598.8	598.8	599.0	599.0			
					619.2	
		641.2				
			643.2			
	645.2					
650.2				649.4		${\mathrm{Cr}}^{3+}{:A}_{2}^{4}\to T_{2}^{4}$
719.4	719.4	719.4	719.2	719.2	719.2	${\mathrm{Cr}}^{3+}{:A}_{2}^{4}\to E^{2}$
741.0	740.6	741.2	741.2			${\mathrm{Nd}}^{3+}{:I}_{9/2}^{4}\to F_{7/2}^{4}{+S}_{3/2}^{4}$
752.0	751.8	752.2	751.8			
763.0	763.0	762.6				${\mathrm{Nd}}^{3+}{:I}_{9/2}^{4}\to F_{7/2}^{4}{+S}_{3/2}^{4}$
792.8	792.8	793.2	793.2			${\mathrm{Nd}}^{3+}{:I}_{9/2}^{4}\to F_{5/2}^{4}{+H}_{9/2}^{2}$
810.4	810.4	810.6	811.2			
817.0	817.0	815.2				${\mathrm{Nd}}^{3+}{:I}_{9/2}^{4}\to F_{5/2}^{4}{+H}_{9/2}^{2}$
835.0	835.0	835.2	835.2			${\mathrm{Nd}}^{3+}{:I}_{9/2}^{4}\to F_{3/2}^{4}$
874.0	874.0	874.2	874.2			${\mathrm{Nd}}^{3+}{:I}_{9/2}^{4}\to F_{3/2}^{4}$
885.6	885.4	885.2	884.6			
898.8	898.6	898.2	897.2			${\mathrm{Nd}}^{3+}{:I}_{9/2}^{4}\to F_{3/2}^{4}$
913.6	913.6	913.4	913.2			
923.6	924.0	924.2	924.2			

**Table 4:** Experimentally observed frequencies in the Raman spectra of LiTaO_3_:Cr:Nd crystals and their assignment.

**Crystal**	**Frequency, cm** ^−1^		**Assignment [70,71,72]**
	$x\left(\mathrm{zz},\mathrm{zy}\right)\;\bar{x}$	$z\left(\mathrm{xx},\mathrm{yy},\mathrm{xy}\right)\;\bar{z}$	
LT-3	140	142	1E(TO)
	182		1E(LO)
	206	205	1A_1_(TO)
	247	250	3E(TO)
	312	309	4E(TO)
	356	353	2A_1_(LO) (3A_1_(TO))
	380	377	5E(TO) (6E(TO))
	461	464	7E(TO)
	515	518	
	595	592	8E(TO) (4A_1_(TO))
	654	660	9E(TO) (8E(LO))
	749	746	
	864	864	9E(LO) (4A_1_(TO))
LT-4	140	146	1E(TO)
	180		1E(LO)
	206	206	1A_1_(TO)
	247	247	3E(TO)
	312	312	4E(TO)
	356	353	2A_1_(LO) (3A_1_(TO))
	377	377	5E(TO)
	461	461	7E(TO)
	521	518	
	595	592	8E(TO) (4A_1_(TO))
	657	660	9E(TO) (8E(LO))
	750	749	
	861	864	9E(LO) (4A_1_(LO))
LT-5	140	143	1E(TO)
		164	
	203	206	1A_1_(TO)
		229	
	250		
		286	
	312	312	4E(TO)
	356	356	2A_1_(LO) (3A_1_(TO))
	377	380	5E(TO) (6E(TO))
	464	464	7E(TO)
	515	510	
	595	595	8E(TO) (4A_1_(TO))
	657	657	9E(TO) (8E(LO))
	749	746	
	861	864	9E(LO) (4A_1_(LO))
LT-6	143	146	1E(TO)
		158	
	186		1E(LO)
	206	206	1A_1_(TO)
	247	250	3E(TO)
	312	309	4E(TO)
	356	353	2A_1_(LO) (3A_1_(TO))
	380	377	5E(TO) (6E(TO))
		412	
	464	461	7E(TO)
	521	515	
	598	595	8E(TO) (4A_1_(TO))
	660	660	9E(TO) (8E(LO))
	745	746	
	864	864	9E(LO) (4A_1_(LO))
LT-7	140	143	1E(TO)
	187		1E(LO)
	206	206	1A_1_(TO)
	250	250	3E(TO)
	315	312	4E(TO)
	356	353	2A_1_(LO) (3A_1_(TO))
	380	377	5E(TO) (6E(TO))
	461	461	7E(TO)
	521		
	595	592	8E(TO) (4A_1_(TO))
	657	660	9E(TO) (8E(LO))
	742	749	
	864	864	9E(LO) (4A_1_(LO))
LT-8	143	143	1E(TO)
	184		1E(LO)
	206	206	1A_1_(TO)
	250	250	3E(TO)
		306	
	312	315	4E(TO)
	356	353	2A_1_(LO) (3A_1_(TO))
	380	377	5E(TO) (6E(TO))
	464	461	7E(TO)
	515		
	595	595	8E(TO) (4A_1_(TO))
	657	657	
	748	746	
	864	864	9E(LO) (4A_1_(LO))

**Table 5:** Values of the concentration of OH^−^ groups [C(OH^−^), cm^−3^], as well as frequencies (ν, cm^−1^), widths (S, cm^−1^) and relative intensities (I, rel. units) of lines corresponding to the stretching vibrations of the OH^−^ groups in the FTIR absorption spectra of LiTaO_3_:Cr^3+^:Nd^3+^ crystals of different compositions.

Crystal	ν	I	S	C (OH^−^ Groups), cm^−3^
LT-1	3459	0.09	6.97	3.90·10^17^
	3462	0.197	13.78	
	3474	0.16	10.01	
	3483	0.06	10.08	
	3493	0.04	16.12	
LT-2	3463	0.05	13.93	4.22·10^17^
	3480	0.15	21.91	
	3493	0.102	18.67	
	3506	0.05	21.16	
LT-3	3463	0.03	13.49	3.73·10^17^
	3478	0.09	21.09	
	3482	0.11	23.85	
	3504	0.05	25.08	
LT-4	3465	0.04	14.29	4.42·10^17^
	3483	0.07	15.74	
	3489	0.13	22.15	
	3505	0.06	21.32	
LT-5	3463	0.12	12.47	10.6·10^17^
	3486	0.23	19.66	
	3489	0.34	25.93	
	3507	0.09	21.80	
LT-6	3462	0.05	10.52	6.03·10^17^
	3486	0.18	22.46	
	3489	0.15	20.38	
	3504	0.10	22.03	
LT-7	3464	0.02	15.93	3.46·10^17^
	3480	0.08	21.42	
	3490	0.10	25.15	
	3508	0.04	28.38	
LT-8	3464	0.02	15.32	3.78·10^17^
	3479	0.07	22.96	
	3493	0.10	27.69	
	3507	0.04	29.19	

## Data Availability

The raw data required to reproduce these findings are available from the corresponding author, A.Yu.P., upon reasonable request.
